# Curcumin-loaded graphene oxide quantum dots enhance otoprotective effects via blocking cuproptosis

**DOI:** 10.3389/fbioe.2023.1183197

**Published:** 2023-04-19

**Authors:** An Hu, Jian-Wei Zhang, Li-Yun Yang, Pei-Pei Qiao, Dan Lu, Ya-Feng Yu

**Affiliations:** ^1^ Department of Otolaryngology, The First Affiliated Hospital of Soochow University, Suzhou, China; ^2^ Department of Otolaryngology-Head and Neck Surgery, Gongli Hospital, Second Military Medical University, Shanghai, China; ^3^ Department of Otolaryngology–Head and Neck Surgery, Huadong Hospital Affiliated to Fudan University, Shanghai, China

**Keywords:** graphene oxide quantum dots, curcumin, ototoxicity, reactive oxygen species, cuproptosis

## Abstract

**Background:** Cisplatin (CIS) is widely used to treat various cancers but can cause ototoxicity and sensory hair cell loss in the inner ear. Copper induces an excessive production of reactive oxygen species (ROS) in hair cells, leading to the development of various antioxidants.

**Methods and results:** This study aimed to evaluate the potential antioxidant properties of curcumin (CUR) in the inner ear organ of corti-1 cells (OC1) and animal models (zebrafish and guinea pigs). Graphene oxide quantum dots (GOQDs) enabled CUR to penetrate the round window membrane (RWM) and maintain the concentration in the perilymph after inner ear administration. The results showed that CUR/GOQDs had favorable biocompatibility and strongly affected ROS generation induced by CIS in OC1 cells. DCFHDA Green staining demonstrated that CUR/GOQDs successfully reversed the decrease in mitochondrial membrane potential induced by CIS *in vitro* and rescued cells from early cuproptosis, which was confirmed by FDX1 staining. Additionally, the experiment found that CUR decreased the expression of cuproptosis proteins (FDX1, LIAS, and LIPT1) and increased the expression of the Bcl-2 protein.

**Conclusion:** The results demonstrate that CUR/GOQDs is a promising therapeutic agent that can prevent CIS-induced ototoxicity by blocking the cuproptosis signal pathway.

## Graphical Abstract

**Figure:**
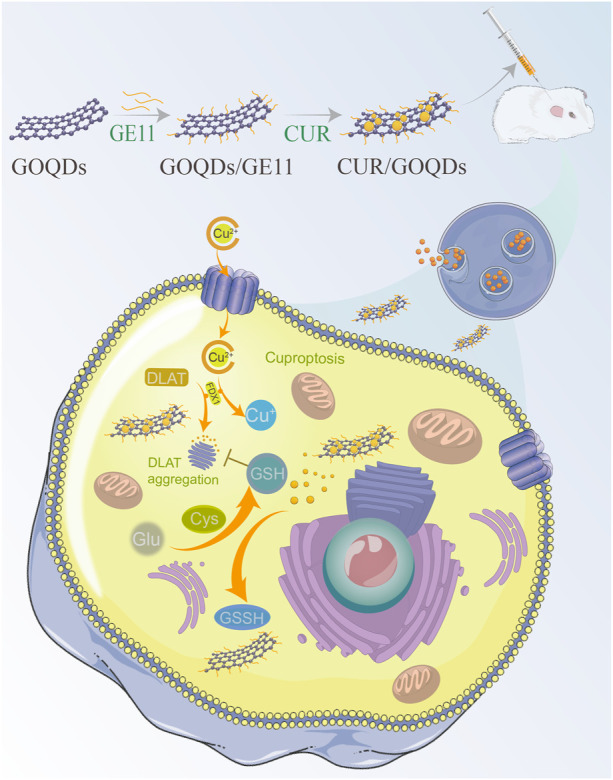
Mechanisms by which exposure to cisplatin disrupts the inner ear system are not yet known. Here, we show that cisplatin exposure is associated with dysregulation of oxidative stress in the inner ear of the rodent. Furthermore, we found that, unlike controls, the response parameters of auditory cells of rats exposed to cisplatin were related to an imbalance in copper metabolism. These data suggest that curcumin related changes in the inner ear are depend on their effects on the mechanism of balance of the copper metabolism and that exposure to cisplatin can disrupt the plastic copper metabolism mechanisms needed to restore normal processing in peripheral auditory cells after hearing loss.

## Background

High-intensity chemotherapy can effectively kill and control tumor cells, but normal tissues are also seriously damaged due to lack of selectivity, resulting in many complications, which greatly affect the treatment plan and effect (Siegel et al., 2022; Fisher et al., 2023). Cisplatin (CIS) has been used in cancer treatment for 40 years (Bejoy et al., 2022; Hyung et al., 2023). Although it has side effects and more targeted therapies have recently been introduced, it is still the pillar of cancer treatment (Peña et al., 2022; Rouprêt et al., 2023). Unfortunately, two side effects of CIS are ototoxicity and nephrotoxicity (Bogart et al., 2022; Doke et al., 2023). Although kidney injury can usually be reversed or prevented, inner ear hair cell injury is permanent. In the case of CIS, up to 63% of treated patients reported permanent hearing loss (Janeway and Grier, 2010). Although ototoxicity is a common consequence of cisplatin chemotherapy, there is little guidance for intervention measures to prevent such permanent and progressive adverse events. The infusion time of cisplatin should not be changed to reduce ototoxicity. It is important to develop methods to prevent hearing loss associated with this drug.

In the cochlea, outer hair cells (OHCs) are more susceptible than inner hair cells (IHCs) (García-Añoveros et al., 2022). OHC death starts from the basal high-frequency end and develops to the apex of the cochlea with longer treatment and higher doses (Kolla et al., 2020). A lower frequency is encoded at the apex of the cochlea. In the presence of copper, it mainly enters through the Cu^2+^ channel at the tips of hair cells (Gourdon et al., 2011). The Cu^2+^ channel effectively acts as a one-way valve to capture Cu^2+^ in cells. Cu^2+^ accumulates rapidly and then triggers a series of events that lead to hair cell death (Tsvetkov et al., 2022). A potential strategy for hearing protection is to identify a competitive Cu^2+^ channel blocker that prevents hair cells from absorbing Cu^2+^ (Overstreet et al., 2022). The exact molecular composition and structure of Cu^2+^ channels are still under debate, so it is difficult to design a specifically effective compound.

Another potential way to prevent hearing loss caused by CIS treatment is to reduce intracellular accumulation of copper-induced reactive oxygen species (ROS) in hair cells to reduce or prevent cuproptosis induction (Kim et al., 2022). Curcumin (CUR), one of the compounds, was found to have excellent properties and protect the hearing of adult rats *in vivo* (Subramaniam et al., 2022). The purpose of this study was to explore the protective mechanism and versatility of the compound (Bagherniya et al., 2022), and to determine whether it has a protective effect against CIS in zebrafish lateral hair cells and organotypic guinea pig cochlear cultured hair cells.

The drug delivery platform based on nanometer material has been widely studied (Cooley et al., 2023) and applied in otoprotective and treatment (Janmohammadi et al., 2023). In inorganic nano carriers, graphene derivatives, such as graphene oxide quantum dots (GOQDs), are less toxic than other inorganic nanomaterials containing heavy metal ions (Yuan et al., 2022). The physiological stability and high drug loading of GOQDs have also been shown (Park et al., 2020), which makes them a promising nanocarrier for drug delivery (Augustine et al., 2021). The importance of targeting delivery has been emphasized due to systemic toxicity caused by non-specific administration (Bodik et al., 2020). Specific targeting of hair cells can significantly reduce the side effects of chemotherapy drugs.

## Results

### Synthesis and characterization of CUR/GOQDs nanoparticles

**FIGURE 1:**
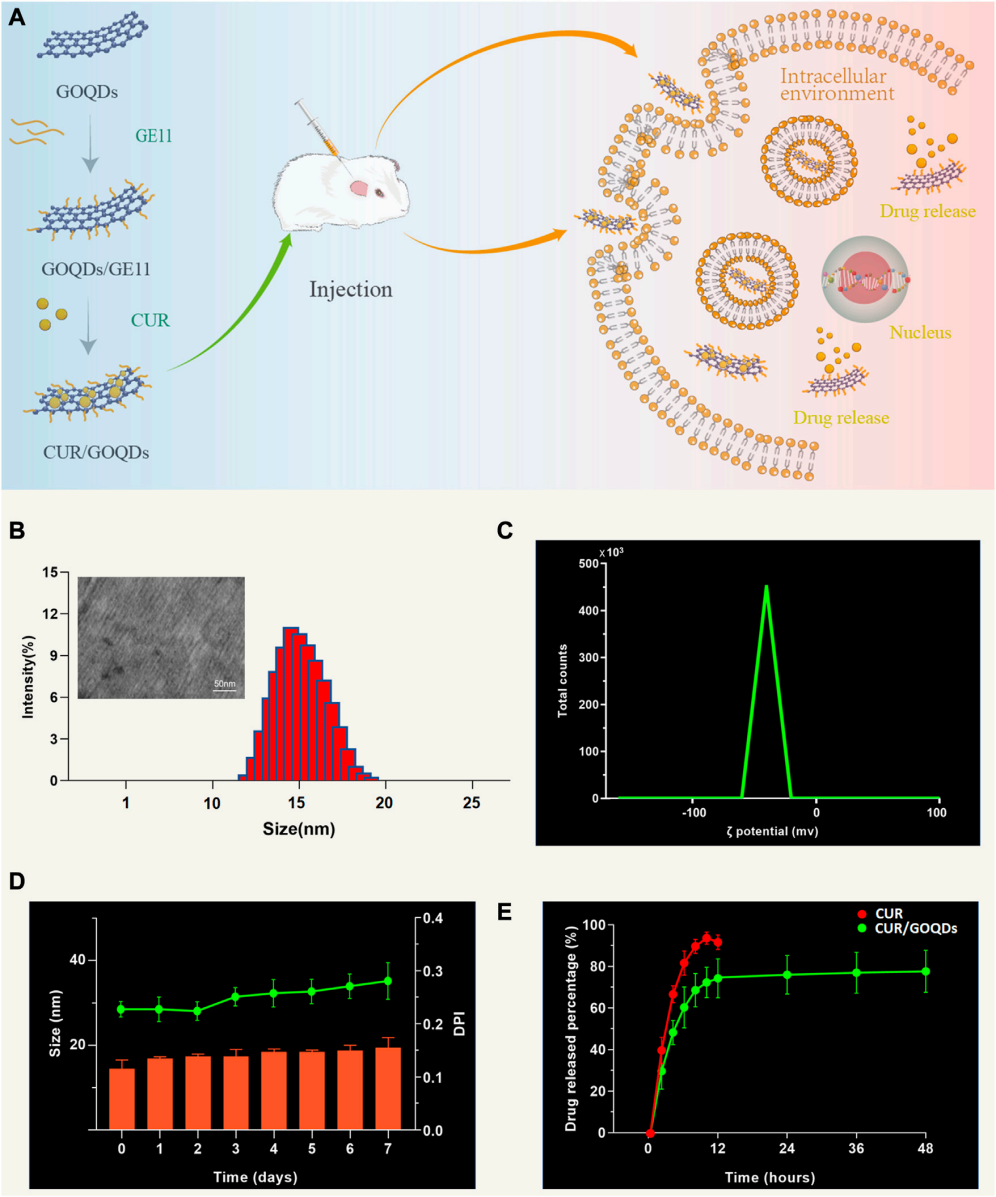
Preparation and characterization of CUR/GOQDs nanoparticles. **(A)** Schematic description of the preparation and structure of CUR/GOQDs. GOQDs were synthesized by an improved Hummer’s method. CUR was transported to cells through the CUR/GOQDs complex to obtain potential otoprotective effects. **(B)** TEM image of CUR/GOQDs and particle distribution (bars in TEM image represent 50 nm). **(C)** The ζ potential of CUR/GOQDs showed a negative charge. **(D)** Particle size and values of PDI at different days. **(E)** Cumulative release curves of free CUR and CUR/GOQDs in cochlea perilymph at different time intervals. (PDI, Polydispersity index; TEM, Transmission electron microscope).

As shown in Figure 1A, this figure shows the microstructure of the prepared CUR/GOQDs nanoparticles. With natural graphite powder as raw material, GO nanosheets were prepared by the modified Hummers method for the first time. Then obtain the GOQDs and modify it to obtain GOQDs@GE11 nanoparticles. CUR is transported to cells through the CUR/GOQDs complex to obtain potential otoprotective. TEM analysis (Figure 1B) shows that the CUR/GOQDs nanoparticles were well diffused and spherical-like without any aggregation. The transmission electron microscope (TEM) image of GOQDs@GE11 showed a regular shape and the diameters of GOQDs@GE11 were 14.32 ± 3.45 nm, the ζ potential of the CUR/GOQDs nanoparticles was approximately −23.4 mV (Figure 1C; Supplementary Table S1). The DL and EE% of SA were 15.12% ± 0.35% and 91.38% ± 2.85%, respectively (Supplementary Table S1), showing an ideal encapsulation effect. To evaluate the stability of CUR/GOQDs nanoparticles, we measured the size and PDI within 7 days. As shown in Figure 1D, the particle size and PDI observed on the third day increased slightly, the size value was still lower than 20 nm and the PDI value was lower than 0.35 until the last day, indicating that the storage stability within 1 week was relatively satisfactory. The CUR release curve of the CUR/GOQDs in AP is shown in Figure 1E. The release curve showed that in the first 12 h, the CUR was released suddenly and about 72.13% of the encapsulated CUR were released from the GOQDs. The CUR release rate then slowed down.

### CUR/GOQDs reduce the cytotoxicity induced by CIS

**FIGURE 2:**
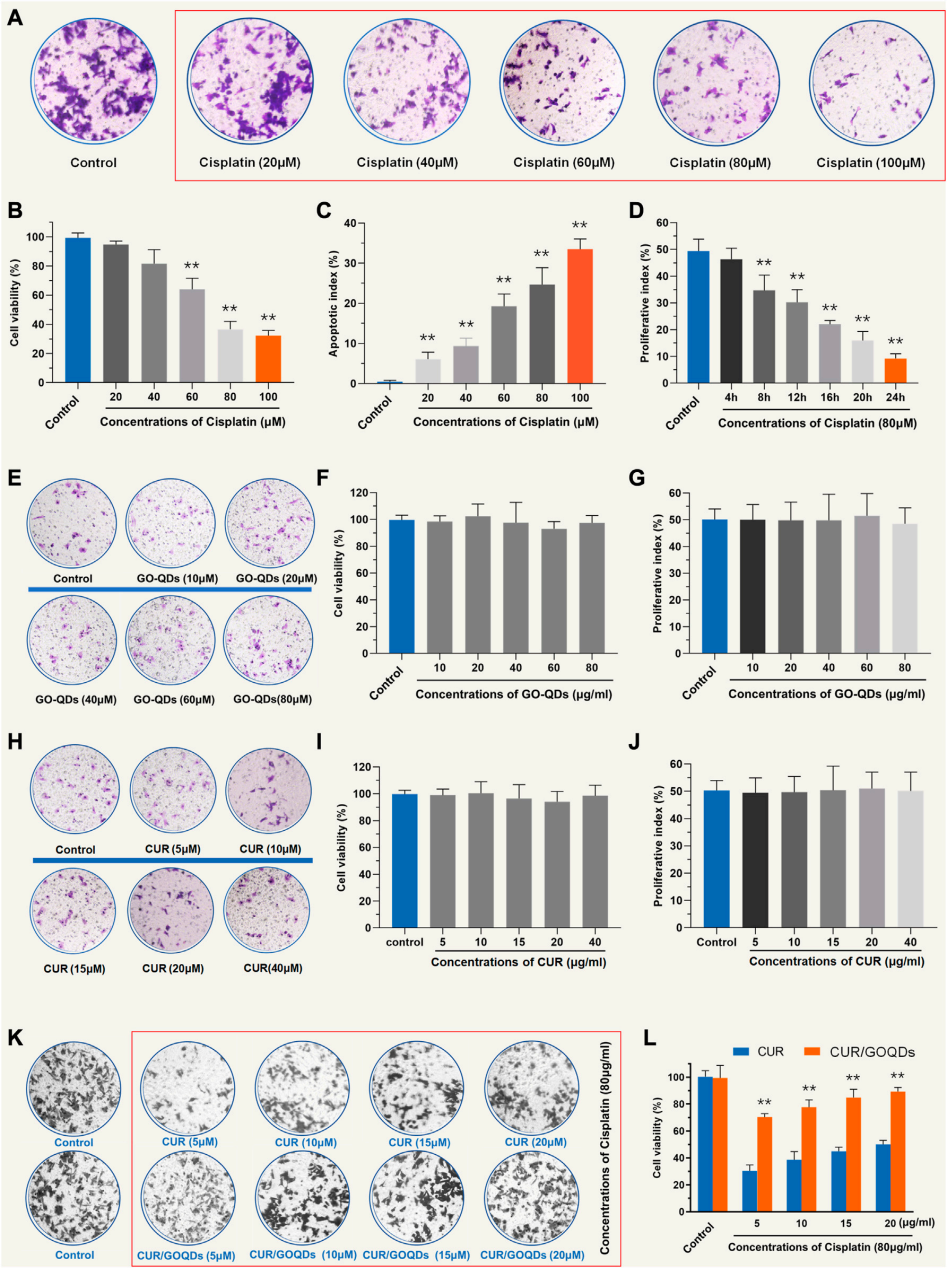
Cell viability of OC1 pretreated with CUR and CUR/GOQDs against CIS-induced cell damage. **(A–C)** Cell viability of OC1 cells treated with a range of concentrations of CIS (20, 40, 60, 80, and 100 μM) for 24 h. **(D)** Survival rate of OC1 cells under 80 μM CIS at different incubation times (4, 8, 12, 16, 20, and 24 h). **(E–G)** The activity of OC1 cells treated with drug-free GOQDs. **(H–J)** In the experiment, additional cell viability tests were conducted for different concentrations of CUR. **(K–L)** Cell viability of OC1 cells before pretreatment with CUR or CUR/GOQDs (5, 10, 15, and 20 μg/mL) followed by treatment with 80 μM CIS for 24 h **p < 0.01 as compared to the control group.

To determine the optimal concentration of CIS to induce OC1 cell injury, we used a series of concentrations (20, 40, 60, 80, and 100 μM) for 24 h. Cell survival rates were 95.56% ± 3.45%, 77.27% ± 12.52%, 58.49% ± 4.24%, 37.68% ± 5.24%, and 30.12% ± 4.65%, respectively (Figures 2A, B). Additionally, we evaluated cell viability at different times (4, 8, 12, 16, 20, and 24 h) after 80 μM CIS treatment (Figure 2C). We chose 80 μM CIS treatment for 24 h as an appropriate condition for OC1 cell injury, because the number of viable cells was reduced to less than 50% of the control (Figure 2D). As shown in Figures 2E–G, no cell ototoxicity of GOQDs was observed at the concentrations of GOQDs studied (10, 20, 40, 60, and 80 μg/mL). We tested whether CUR has cytotoxicity to normal OC1 cells. In the experiment, additional cell viability tests were conducted for different concentrations of CUR (Figures 2H–J). We also tested the cytoprotective effects of CUR/GOQDs against CIS damage at different concentrations (5, 10, 15, and 20 μg/mL). The cell activity results showed that at the experimental concentration (5, 10, 15, and 20 μg/mL) the percentage of living cells in CUR/GOQDs plus CIS were 71.59% ± 9.87%, 75.78% ± 11.15%, 83.47% ± 9.58%, and 90.25% ± 8.12% (Figures 2K, L). However, the CUR + CIS (5, 10, 15, and 20 μg/mL) groups showed no improvement in viable cells. In conclusion, these results suggest that CUR/GOQDs can attenuate CIS-induced cytotoxicity in OC1 at experimental concentrations.

### Cellular uptake of CUR/GOQDs

**FIGURE 3:**
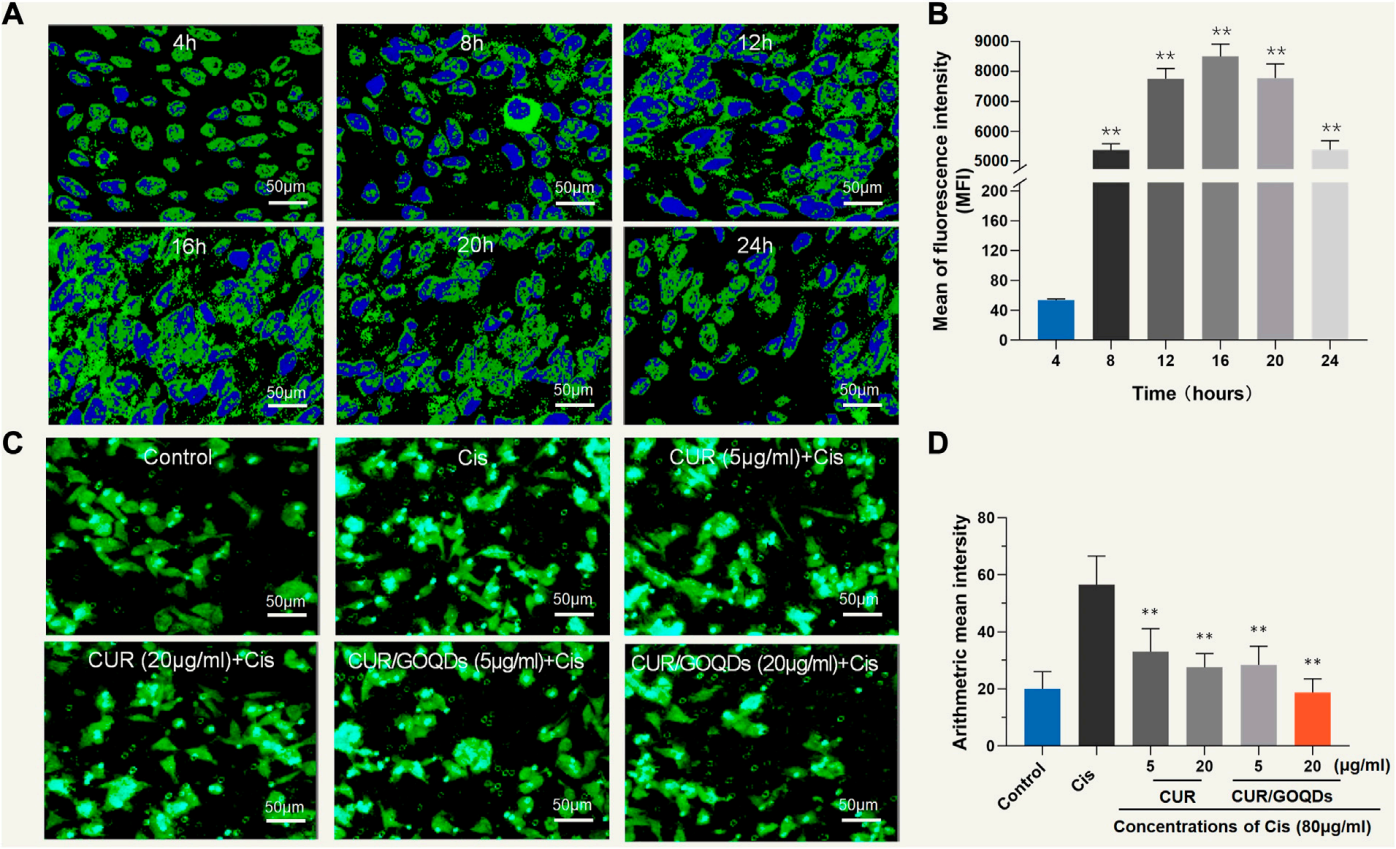
Cellular uptake of CUR/GOQDs in OC1 cells. **(A)** Confocal images of internalization in different periods. **(B)** The average fluorescence intensity of OC1 cells was measured by flow cytometry. **(C)** ROS level detection (DCFHDA) pretreated with CUR or CUR/GOQDs (5 and 20 μg/mL) for 6 h, followed by CIS (80 μM, 12 h). Confocal images showing DCFHDA stained intracellular ROS (green fluorescence) increased in OC1 cells treated with 80 μM CIS and was significantly inhibited by CUR or CUR/GOQDs. **(D)** Quantification of the average fluorescence intensity. ROS decreased significantly after the application of CUR or CUR/GOQDs. **p < 0.01.

As shown in Figures 3A, B, CUR/GOQDs were easily internalized by OC1 cells after 4 h of incubation. Furthermore, cell uptake increased with prolongation of culture time, reached its peak at 16 h and then decreased (*p* < 0.01). CUR/GOQDs can reduce CIS-induced mitochondrial dysfunction and oxidative damage. Previous studies have confirmed that CIS induces oxidative damage by triggering the accumulation of reactive oxygen species. Therefore, we investigated the oxidative status of OC1 cells treated with CIS. Intracellular ROS levels were monitored by the fluorescent probe DCFHDA. As demonstrated in Figures 3C, D, significant accumulation of ROS caused by CIS (80 μM, 12 h), as shown by green fluorescence in cells. However, pretreatment with CUR or CUR/GOQDs (5 or 20 μg/mL) could inhibit the generation of ROS induced by CIS. The statistical results further confirmed the antioxidant effect of CUR and CUR/GOQDs.

### CUR/GOQDs alleviates CIS-induced cuproptosis

**FIGURE 4:**
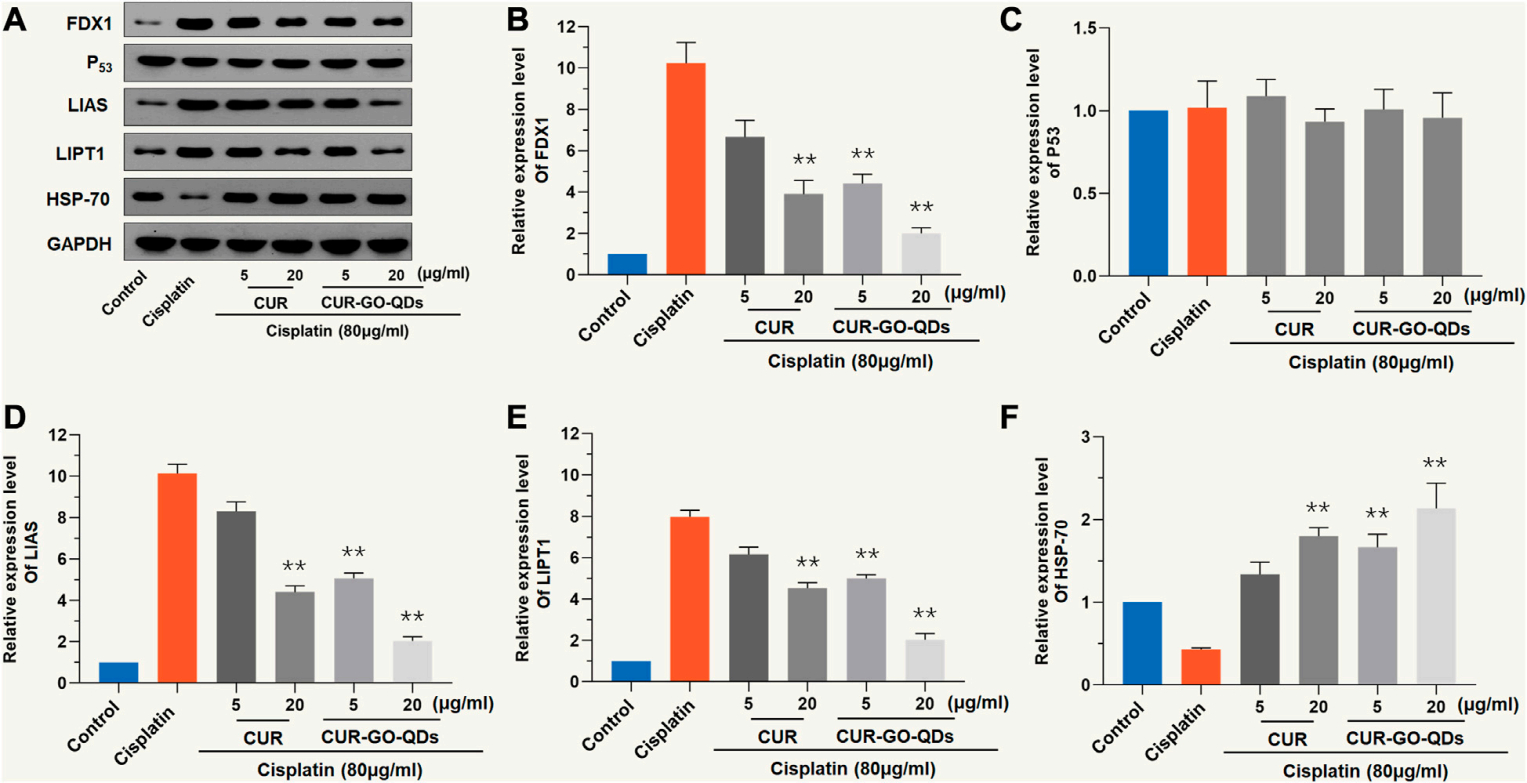
CUR/GOQDs alleviates CIS-induced cuproptosis. CIS (80 μM, 24 h) induced cuproptosis of OC1 cells after pretreatment with CUR, or CUR/GOQDs (5 and 15 μg/mL) for 4 h. **(A)**Western blot with anti-FDX1, anti-P53, anti-LIAS, anti-LIPT1 and anti-HSP-70 antibodies. **(B–F)** Quantification of Western blot in **(A)**. **p < 0.01, as compared to CIS.

As shown in Figures 4A–E, CIS treatment significantly increased the protein expression levels of pro-cuproptotic protein, FDX1, LIAS, and LIPT1, but pretreatment with CUR (5 μg/mL) and CUR/GOQDs (5 or 15 μg/mL) significantly inhibited the expression levels of these proteins. Furthermore, the expression level of the HSP-70 protein decreased after CIS (80 μM, 24 h) treatment and increased after pretreatment with CUR (5 or 15 μg/mL) and CUR/GOQDs (5 or 15 μg/mL) (Figure 4F).

### CUR/GOQDs attenuates the CIS-induced activity of DLAT

**FIGURE 5:**
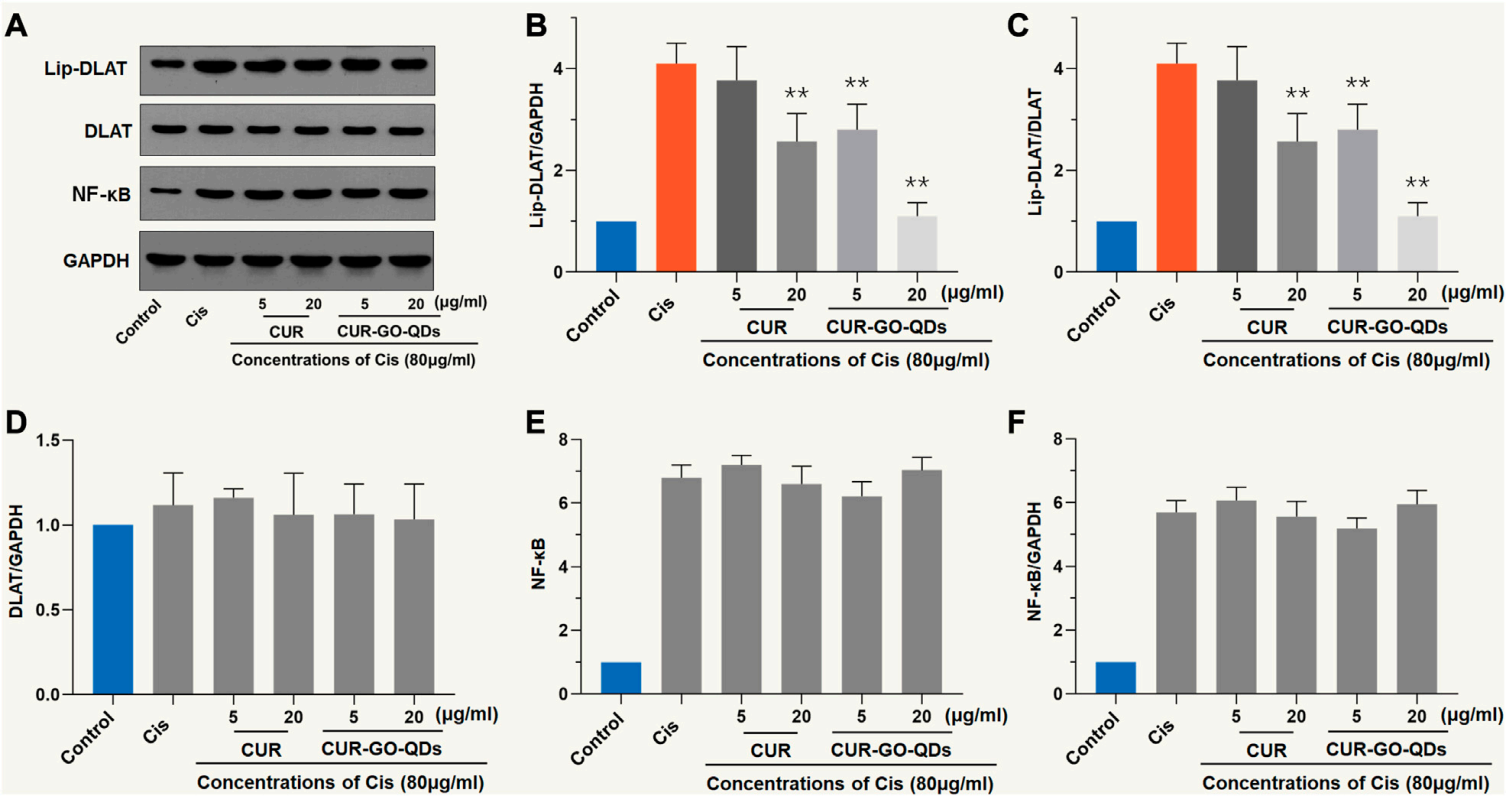
Western blot analysis with DLAT. **(A)** OC1 cells were treated with CIS and CUR or CUR/GOQDs as described above. Detection of DLAT expression by WB. **(B–D)** The light intensity of protein expression was quantified according to the above WB results. The application of CUR or CUR/GOQDs in OC1 treated with CIS resulted in a decrease in the ratio of Lip-DLAT/GAPDH **(B)** and Lip-DLAT **(C)**, while the ratio of DLAT **(D)**, NF-κB **(E)** and NF-κB/GAPDH **(F)** did not change. **p < 0.01, as compared to CIS.

As shown in Figures 5A–C, CIS (80 μM, 24 h) activated DLAT, characterized by an increase in the degree of lipoacylation in total DLAT. However, the anti-lipoacylation effect of 5 or 15 μg/mL CUR or CUR/GOQDs on OC1 cells treated with CIS was quite different (Figure 5B), which is reflected in the significant decrease in the ratio of Lip-DLAT and Lip-DLAT/GAPDH, while nearly no change was observed in the DLAT, NF-κB and NF-κB/GAPDH ratios (Figures 5D, F). More specifically, 15 μg/mL CUR/GOQDs reduced FDX1 activity more effectively than 5 μg/mL CUR (*p* < 0.05).

### Protective effect of CUR/GOQDs on zebrafish hair cell injury

**FIGURE 6:**
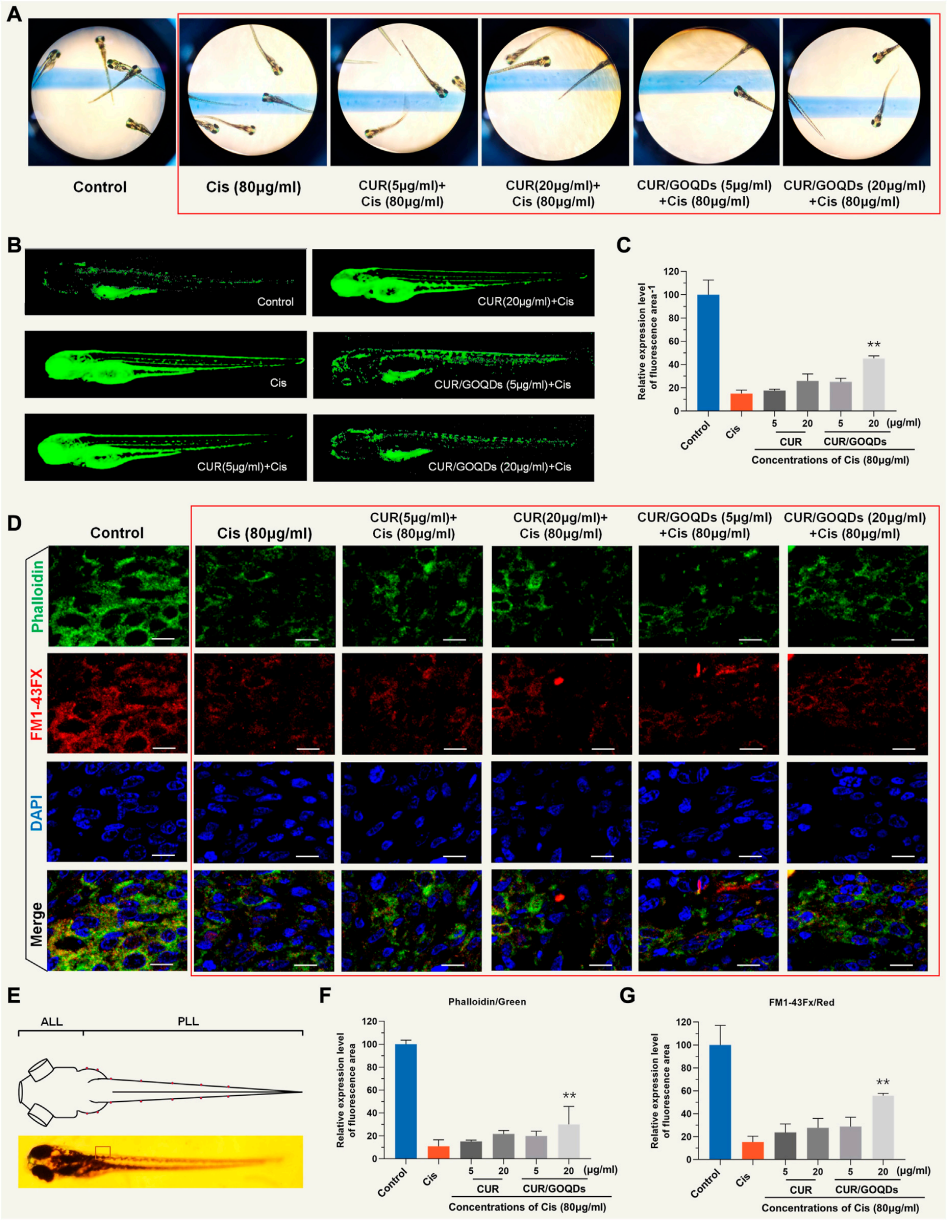
Evaluation of hair cells damage by DASEPI in zebrafish. **(A–B)** Zebrafish hair cells stained with DASEPI (green) showed the damage to CIS and the protective effect of CUR or CUR/GOQDs. **(C)** Calculated the percentage of protection of CUR or CUR/GOQDs. **p < 0.001. **(D)** Hair cells, stereocilia, and nuclei were stained with FM1-43FX (red), phalloidin (green), and DAPI (blue), respectively. The dorsal view of zebrafish larvae shows the distribution of hair cells along the body **(E)**. Anterior lateral line (ALL) and posterior lateral line (PLL). **(F–G)** The number of hair cells of the zebrafish larvae decreased significantly treated with CIS. Scale bars = 10 μm.

We evaluated neuronal hair cell injury by measuring the fluorescence intensity of DASPEI and calculating the protective effect (Figure 6A). Both CUR and CUR/GOQDs at concentrations of 5 and 15 μg/mL could not reduce the damage to hair cells induced by CIS (Figure 6B). Only when the concentration was 20 μg/mL CUR/GOQDs had a significant protective effect on zebrafish hair cell damage (35%) (Figure 6C). As shown in Figures 6D, E, zebrafish larvae were then labeled with FM1-43FX to evaluate the effect on the number of hair cells in the anterior lateral line (ALL) and posterior lateral line (PLL). As shown in Figure 6D, the number of hair cells of the zebrafish larvae decreased significantly. Furthermore, the zebrafish larvae were also stained with phalloidin to assess the integrity of the lateral hair cells. The results were consistent with FM1-43FX staining (Figures 6F, G).

### CUR/GOQDs partially restored CIS-induced hearing loss in Guinea pigs

**FIGURE 7:**
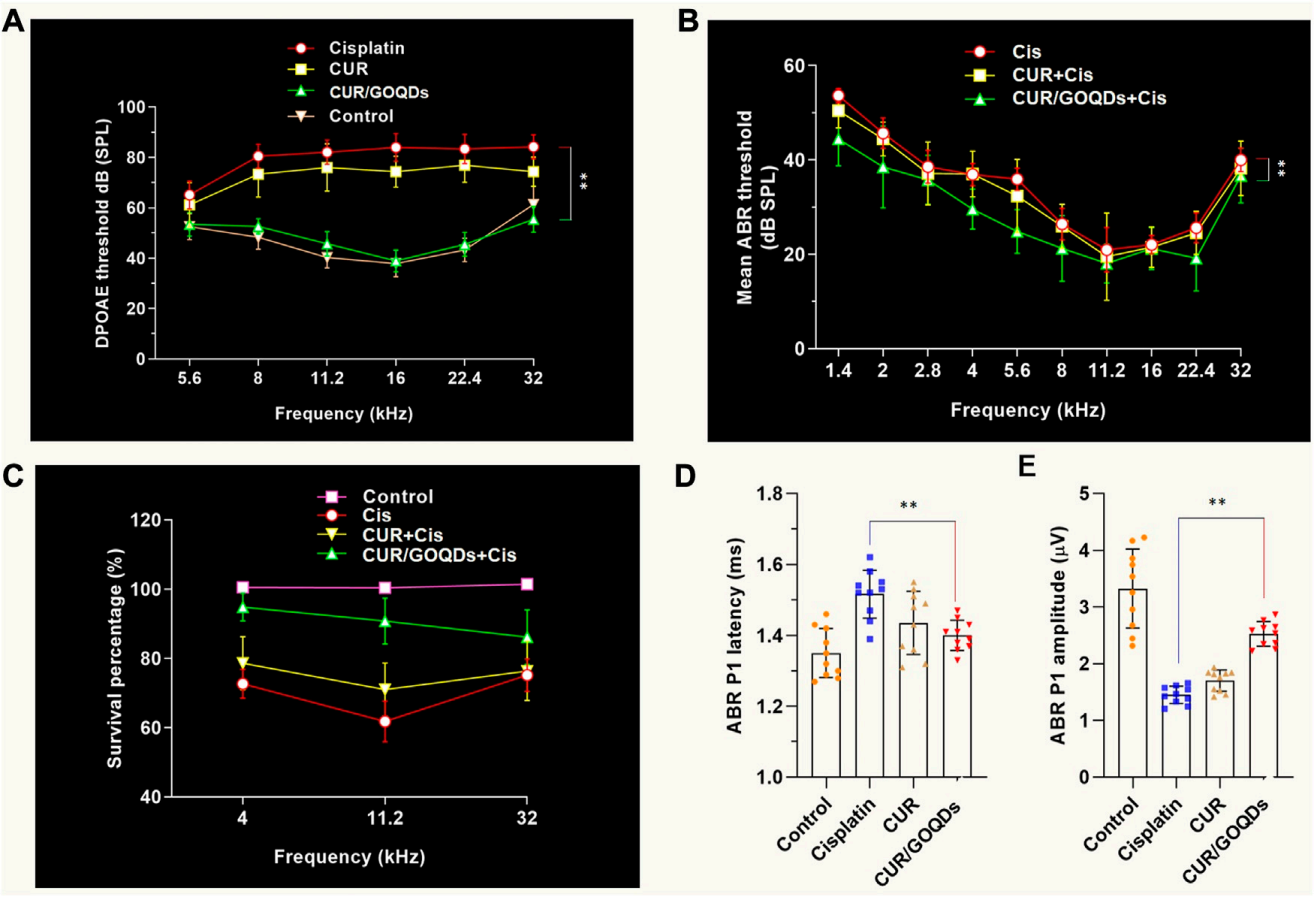
CUR/GOQDs partially rescue CIS-induced hearing loss. **(A–C)** High concentration of CIS causes severe hearing loss inguinea pigs. Guinea pigs treated with CIS of different concentrations for 2 weeks were tested according to DPOAE and ABR. Mean ABR threshold of guinea pigs treated with CIS, CUR or CUR/GOQDs. **(A)** DPOAE threshold and ABR threshold, **(B)** ABR peak 1 (P1) latency, **(C)** ABR peak 1 (P1) amplitude. **(D–E)** The DPOAE and ABR thresholds were significantly higher in the high-dose (80 mg/kg) CIS treated group than in the untreated group; *n* = 10.

Determine the most effective dose and the least side effects of CIS in the auditory system and then evaluate the efficacy of different CUR/GOQDs treatments on guinea pig hearing. It should be noted that the intensity of the distortion product otoacoustic emission (DPOAE) of guinea pigs injected with high-dose CIS decreased significantly. Additionally, injection of high-dose CIS (80 mg/kg) for 2 weeks resulted in severe hearing loss in guinea pigs (Figure 7A). In guinea pigs injected with high concentration CIS, the ABR threshold between 1.4 and 32 kHz stimulation significantly increased the sound pressure level (SPL) by about 10 dB–20 dB. The latency of ABR wave I increased (Figure 7B) and the amplitude of the peak of ABR wave 1 (P1) decreased at the same time (Figure 7C), highlighting the auditory defects of guinea pigs injected with different drugs. *In vivo* electrophysiological data showed that hearing impairment in guinea pigs given high-dose CIS resulted from impaired peripheral auditory signal processing (Figures 7D, E).

### CUR/GOQDs effectively reversed CIS-induced hair cell loss

**FIGURE 8:**
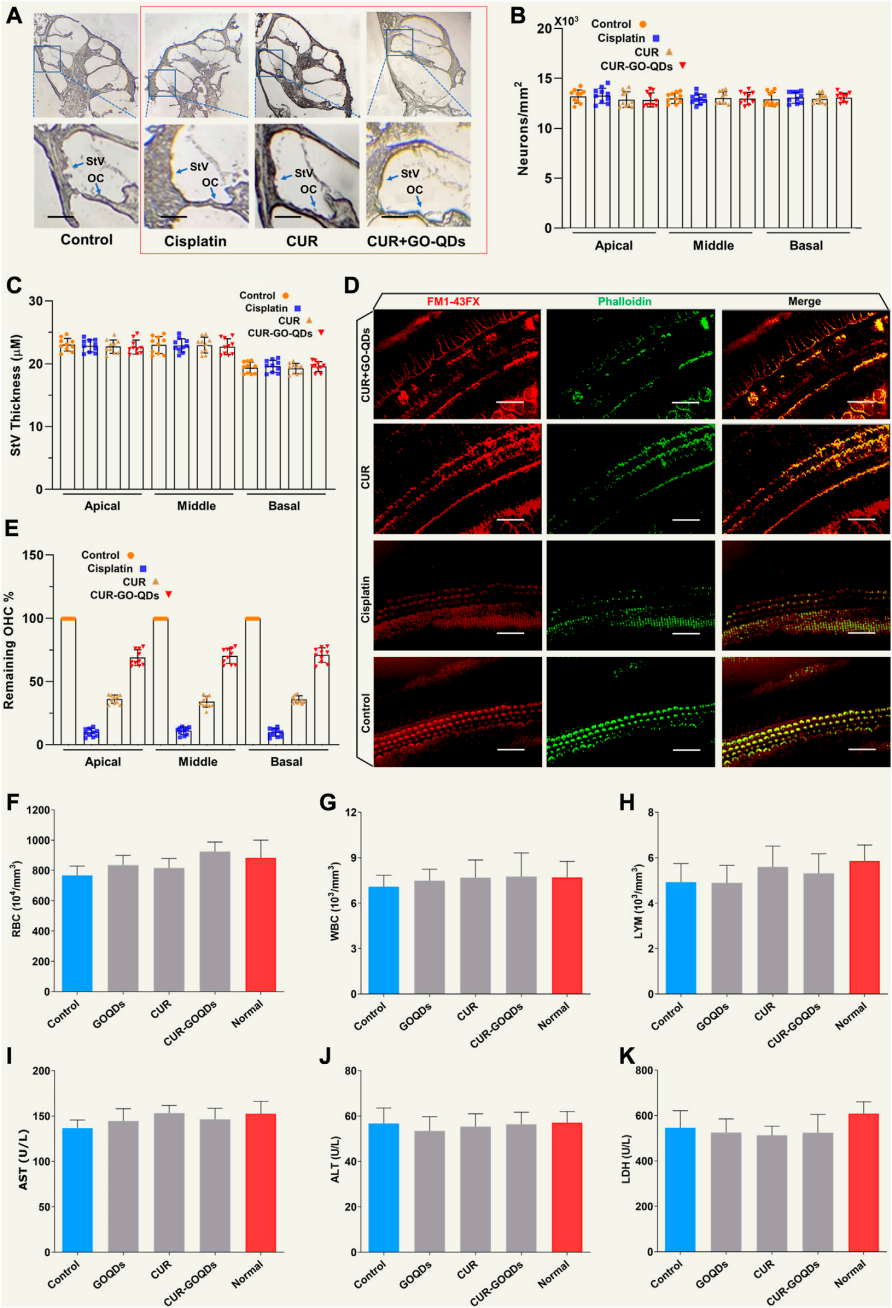
CUR/GOQDs partially restored CIS-induced hearing loss in inguinea pigs. **(A)** H&E staining was performed on the transverse section of the guinea pig cochlea treated with different CIS. **(B)** Severe degeneration of HCs was observed in the OC (Corti organ) in guinea pigs treated with high-dose CIS, but not in the control group and CUR/GOQDs group. However, there were no significant differences in SGN and StV among the three groups. The survival pattern and StV thickness **(C)** of ganglion cells were similar among the four groups. **(D)** *In vitro* culture of Corti organs. Phallioidin and FM1-43FX staining images of the cultured cochlear sensory epithelium treated with CIS. **(E)** By analyzing the number of cochlear revolutions in different treatment groups, the OHCs that survived after CIS treatment were quantitatively analyzed; *n* = 10. (Scale bar: 20 µm) Peripheral blood was collected in the presence of the anticoagulant EDTA and analyzed using a hematological analyzer. Red blood cells **(F)**, white blood cells **(G)**, and lymphocytes **(H)** were analyzed separately. Peripheral blood cell analysis showed no significant decrease in lymphocytes in the CUR-GOQDs treatment groups compared to normal guinea pigs **(I–K)**. Compared to the control group, the levels of AST, ALT, and LDH in the treated rats slightly decreased. Data are presented as mean ± SD, **p < 0.01, using two-tailed Student’s *t*-test.

To study the potential causes of hearing, we found by histopathology that a high concentration of CIS led to severe hair cell loss, while there was no significant change in other key areas of the cochlea, such as spiral ganglion neurons (SGN) and stria vascularis (StV) (Figures 8A–C). Consistent with the phenotype of the living cochlea, we observed a significant loss of IHCs (inner hair cells) and OHCs (outer hair cells) with CIS *ex vitro* (Figure 8D). CUR/GOQDs can reduce CIS-induced OHCs damage, and the damage to the basal basilar membrane was significantly different from that of the middle membrane (Figure 8E). Blood parameters were measured after drug administration to investigate whether CUR-GOQDs treatments had any side effects on guinea pigs. Blood tests conducted 3 days after drug injection showed some changes in hematological parameters. Compared to the control group, the red blood cell, white blood cell, and lymphocyte counts of the treated guinea pigs did not increase significantly (Figures 8F–H). Hematological analysis of peripheral blood showed no significant decrease in AST, ALT, and LDH levels in the experimental guinea pigs compared to normal guinea pigs (Figures 8I–K). In general, our *in vivo* and *in vitro* results show that different drug treatments have different effects on auditory HCS and that high concentrations of CIS are harmful to guinea pig hearing.

## Discussion

CIS was an antitumor drug, which was indispensable in the clinical setting (Morizane et al., 2022). However, ototoxicity usually limits its wide application (Hoimes et al., 2022). Considerable efforts have been made to explore new drugs to reduce or prevent CIS-related ototoxicity (Ho et al., 2022). Although no drugs have been approved to be completely effective to date (Salaroglio et al., 2022), many drugs have achieved success in preclinical work and some of them have shown protective effects in the clinic (Brown et al., 2022). In this study, we established GOQDs for loading CUR to successfully promote penetration of RWM and maintained CUR concentration in the inner ear perilymph for 24 h after a single injection. CUR/GOQDs have favorable biocompatibility and strongly affect CIS induced ROS production in OC1 cells. In addition, CUR/GOQDs successfully reduced the loss of OHCs in animal models (zebrafish and guinea pigs).

CIS has great potential as a new type of cancer therapy (Squillace et al., 2022). Studies have shown that its effective anticancer activity was due to cuproptosis caused by elevated levels of reactive oxygen species (Goldberg et al., 2022). We have previously performed experiments with isolated mitochondria without copper, thus hindering the identification of the essential role of this ion. Some studies have deciphered the mechanism of ROS induced by FDX1 in cancer cells (Britti et al., 2021), which is a unique mode of action dependent on its copper chelating ability. After cellular uptake, the FDX1-Cu complex selectively and rapidly targets mitochondria. In this organelle, Cu^2+^ was oxidized and reduced to Cu^1+^, resulting in oxidative stress. Importantly, after separation from the complex, the outflow of FDX1 promotes the repeated shuttle of the FDX1-Cu complex to promote the accumulation of copper and the production of ROS sufficient to cause cell death.

CIS induced ototoxicity usually occurs in the early stage, mainly affects the high frequency, and leads to progressive and cumulative hearing loss (Baba et al., 2012). As previously reported, DPOAE and ABR were dysregulated in guinea pigs affected by CIS (Jamesdaniel et al., 2016). Our research has shown that CUR/GOQDs could improve the degenerative auditory response, ranging from 4 to 32 kHz. Measurement of distortion product otoacoustic emissions (DPOAEs) and auditory brain stem response (ABR) showed that auditory function of guinea pigs injected with CIS intraperitoneally was impaired, and CUR/GOQDs injected into the left ear through the tympanic cavity could partially prevent loss of auditory function. In terms of mechanism, downregulation of B-cell Lymphoma 2 (Bcl-2) expression and upregulation of FDX1 expression were detected in OC1 cells and cochlear explants exposed to CIS, which can be prevented by pretreatment with CUR/GOQDs. Our study shows that CUR/GOQDs pretreatment can reduce CIS-induced ototoxicity by inhibiting the cuproptosis signal pathway.

## Conclusion

The CUR/GOQDs were screened from newly synthesized compounds (Chae et al., 2018) due to their high otoprotective efficacy and good pharmacokinetic characteristics (Ghasemlou et al., 2022). Furthermore, it did not interfere with the anticancer effect of CIS. This study supplements the evidence that CUR/GOQDs can effectively protect the hearing of guinea pigs after administration (Abd-Elhakim et al., 2022). It is well tolerated and does not affect hearing itself, even if the daily dose concentration is several weeks higher than the concentration used for ear protection. Although the detailed mechanisms must be further investigated, the results suggest that CUR/GOQDs may be a potential agent to attenuate CIS-induced hearing loss. The next step to bring these findings to the clinic involves confirming that these favorable characteristics also apply to human subjects.

## Materials and methods

### Cell culture

The OC1 cell line (Organ of Corti-1 cells produced by the House Ear Institute, United States) was cultured in Dulbecco’s modified Eagle medium supplemented with 10% fetal bovine serum at 37°C under an atmosphere of 5% CO_2_. Install each hole 5×10^4^ cells were inoculated according to the manufacturer’s instructions on 24-well plates coated with fibronectin and the proliferation test was performed. Each case was evaluated in triplicate.

### Animal experiments

Animals without otitis media (guinea pigs 4–6 weeks old, weighing 0.2–0.25 kg) were purchased from the Shanghai Laboratory Animal Center (Shanghai, People’s Republic of China). All animals were raised by the Animal Resource Facility of the First Affiliated Hospital of Soochow University. Animal experiments were carried out in strict accordance with the “principles for the care and use of vertebrates” and “guidelines for the use and care of experimental animals”, and were approved by the ethics committee of experimental animals of the First Affiliated Hospital of Soochow University. Guinea pigs were exposed to 92% N_2_/8% O_2_ in a closed plastic room at 1 atmospheric pressure. The plastic room is humidified and ventilated to minimize pCO_2_ change. At the end of the experiment, the guinea pigs were killed with an excess of sodium pentobarbital (4%, 200 mg/kg by intraperitoneal injection; Sigma, Shanghai, China).

### Synthesis of GOQDs

GOQDs were obtained by ultrasonic stripping. GO was dispersed in DMF (N, N-dimethylformamide) at a concentration of 15 mg/mL and the mixture was sonicated for 45 min (115 W, 125 kHz). The mixed solution was then transferred to a 35 mL autoclave lined with polytetrafluoroethylene and heated at 180°C for 3 h. Then the container was cooled to 30°C with water, the black sediment was collected, rinsed with water, and then suspended in PBS for later use.

### Preparation of CUR-laden graphene oxide quantum dots (CUR/GOQDs)

In order to prepare CUR-laden complexes, CUR was added to the GOQDs solution. In summary, 6 mg of CUR-HCl and 15 mg of GOQDs were dissolved in distilled water and 10 μL triethylamine was added to the mixture solution. Finally, 5 mL of CUR (5 mg/mL, DMSO) solution was added to the mixing system and the reaction was stirred overnight. Then, the solution was dialyzed and lyophilized to obtain a CUR/GOQDs complex. The loading amount of CUR in the CUR/GOQDs complex was determined by UV-vis spectrophotometry and HPLC.

### Characterization of CUR/GOQDs

The shape, size, and morphology of the synthesized nanoprobe were studied by transmission electron microscopy at 120 kV and the Zetasizer Nano ZS (Malvern, United Kingdom) apparatus. Fourier transform infrared spectra of all samples were collected in transmission mode using KBr plate on a PerkinElmer spectrum 100 FT-IR spectrometer. Analysis of confocal optical micrographs by a confocal laser scanning microscope.

### *In vitro* drug release

The CUR/GOQDs dispersion was placed in a dialysis bag, then immersed in a release vessel containing PBS (pH 5.2, 0.2 M) and stirred in the dark at 37°C. 2 mL of sustained release buffer was collected from each container at different time intervals and replaced with fresh PBS. To measure the drug release in each time interval, the collected samples were analyzed by high performance liquid chromatography and UV-vis spectroscopy.

### Cell viability assays

*In vitro* cytotoxicity evaluation of nanoparticles using the CCK-8 assay. The density of 6×10^4^ OC1 cells/well were inoculated in 48-well plates and adhered overnight. They were then divided into three groups: different concentrations (5, 10, 15, and 20 μg/mL) of CUR or CUR/GOQDs; CIS (20, 40, 60, 80, and 100 μM); and cotreatment with CIS (80 μM) and CUR or CUR/GOQDs (5, 10, 15, and 20 μg/mL). After 24 h of incubation, 10 μL CCK-8 reagent was added to each well and reacted in 5% CO_2_ for 2 h at 37°C. Measurement of absorbance at 450 nm with a flat panel reader. The percentage of cell viability was calculated by comparing the cells treated with different formulas with the corresponding control cells.

### Measurement of ROS induction

To measure superoxide in intact cells, OC1 cells were cultured for 3 h in the presence of 80 nM CIS or control (DMSO). Cells were washed in Hanks buffer at 37°C and stained with 5 mM MitoSOX red superoxide indicator (Invitrogen, United States) for 10 min. In the presence of 12.5 mM glutamate, malate, and pyruvate, the effect of the copper complex on mitochondrial ROS production was studied; ROS-dependent fluorescence was excited at 535 nm and read at 585 nm.

### Copper assays

After three washes in PBS, the cell pellets were prepared and weighed. Maxxam Analytics (Burnaby, BC, Canada) analyzed the copper content in cells by inductively coupled plasma mass spectrometry (ICP-MS). In the tracer experiment, OC1 cells were enriched in medium containing 4 mM CuCl_2_ for 120 h. Then, copper-rich OC1 cells were cultured with 80 mM CIS in a medium without copper addition or in the presence of CuCl_2_. The copper content was also determined photometrically using the copper-selective indicator bicinchoninic acid. In summary, copper was extracted in 285 mM hydrochloric acid before deproteinization with trichloroacetic acid. The total copper content was determined in the presence of a reducing agent (ascorbic acid) and the spontaneous release of copper was determined in the absence of a reducing agent.

### Western blotting

To determine protein level in the cuproptosis pathway, OC1 cells were treated with 80 μM CIS and 5 or 20 μg/mL CUR/GOQDs as described above, and then the cells were collected for Western blot analysis. The protein extract was electrotransferred to the PVDF membrane. The membrane was then blocked in a rapid blocker at room temperature for 1 h and incubated overnight in a cold chamber (4°C) with specific primary antibodies. After incubation, the membrane was cleaned with TBS and then incubated with HRP-binding secondary antibody at room temperature for 1 h, washed repeatedly, and observed with an enhanced chemiluminescence kit. GAPDH as an internal standard.

### ABR experiments

The ABR of guinea pigs aged 30–40 days was measured before and 2 weeks after drug treatment. In summary, guinea pigs were subcutaneously injected with 80 mg/kg/d CIS every day for 10 consecutive days, and the protective effects of three doses of CUR/GOQDs [5, 15 or 20 mg/kg/d] were evaluated. The control group included GOQDs (15 mg/kg/day) and normal saline alone. All guinea pigs survived for 2 weeks after drug treatment without additional drug exposure.

### Zebrafish

The Zebrafish were immersed in the Tricaine anesthetic and then fixed in 0.1 M PBS (pH 7.4) with 4% paraformaldehyde at room temperature for 1 h, and blocked with PBS supplemented with 0.1% triton X-100% and 5% normal goat serum at 25°C for 1–2 h. Samples were cultured overnight at 4°C in 1:400 primary antibody, and then combined with Alexa fluor at 4°C in 1:500 secondary antibody, and washed with PBS +0.1% Triton X-100 between all steps.

### Cochlear cultures

Test CUR/GOQDs at each concentration in at least 3 cochlear cultures. Application ×40 objective lens (0.75 NA) was used to capture the image of labeled hair cells on the microscope from approximately 20% of the middle apical and middle basal regions of the top and bottom of the cochlea, respectively. The corresponding characteristic frequencies of the mature guinea pig cochlea were calculated at these locations and were found to be 45 and 8.5 kHz, respectively. Cell counts in CIS-treated medium were based on the presence of hair bundles and cell bodies. Since CIS-induced damage resulted in approximately 11% of cells which means that hair bundles were independent of the cell body, CIS-treated cultures were quantified this way.

### Blood sample analysis

Blood samples are analyzed by measuring hematological parameters, including red blood cells, white blood cells, and lymphocytes, using an automated cell counter. To determine clinical chemistry parameters, blood samples are collected in microtubes and serum is separated by centrifugation. Glutamic-oxaloacetic transaminase (GOT, AST), glutamic-pyruvic transaminase (GPT, ALT), and lactate dehydrogenase (LDH) are measured using an automatic analyzer.

### Statistics

SPSS 13.0 was used for two-tailed *t*-test (to assess the effects of CUR/GOQDs alone) or one-way or two-way ANOVA (for all other comparisons). When significant main effects and/or interactions were found, Sidak, Dunnett, or Tukey *post hoc* tests were used for appropriate individual comparisons. For graphical display, the data were normalized to the untreated control groups so that 100% represents the hair cell survival of the control animals. A one way ANOVA was performed on the primitive cell count of cochlear culture using GraphPad Prism 8.0, and a Sidak *post hoc* test was performed. If it was found that the cell count of the treatment was significantly different from that of CIS alone, and there was no significant difference with that of the control, it will be classified as a completely protective treatment; If it was significantly different from CIS alone and the control group, it was considered that the treatment has a partial protective effect; If the treatment was significantly different from the negative control group, but there was no significant difference with the simple CIS control group, it was considered that the treatment has no protective effect. 2-way mixed ANOVA and Dunnett’s post-tests were used to evaluate the difference between threshold shift and pretreatment value of ABR records at 2, 4, 8, 16, and 32 kHz. The result was considered significant when *p* ≤ 0.05, the level of statistical significance was shown in the figure.

## Data Availability

The original contributions presented in the study are included in the article/supplementary material, further inquiries can be directed to the corresponding author.

## Abbreviations

ABR, auditory brainstem response; ALL, Anterior lateral line; CIS, cisplatin; CUR, curcumin; DASPEI, 2-(4-(dimethylamino)styryl)-1-ethylpyridinium iodide; DLAT, dihydrolipoyl transacetylase; DPOAE, distortion product otoacoustic emission; FDX1, ferredoxin 1; GOQDs, graphene oxide quantum dots; HSP-70, heat shock protein 70; ICP-MS, inductively coupled plasma mass spectrometry; IHCs, inner hair cells; LIAS, lipoic acid synthase; LIPT1, lipoyltransferase 1; OC1, organ of corti-1 cells; OHCs, outer hair cells; PLL, posterior lateral line; ROS, reactive oxygen species; RWM, round window membrane; SGNs, spiral ganglion neurons; SPL, sound pressure level; StV, stria vascularis; TEM, transmission electron microscope.
