# Supplementary material for: Curcumin-loaded graphene oxide quantum dots enhance otoprotective effects via blocking cuproptosis
Source: Front Bioeng Biotechnol. 2023 Apr 19;11:1183197. doi: 10.3389/fbioe.2023.1183197 (PMC10154464; doi:10.3389/fbioe.2023.1183197)
Supplement: Supplementary file 1 [file Table1.docx]

Table S1. Characterization of CUR/GOQDs nanoparticles.

| **Samples** | **Size (nm)** | **PDI** | **ζ potential (mV)** | **EE (%)** | **DL (%)** |
| --- | --- | --- | --- | --- | --- |
| GOQDs | 14.32±3.45 | 0.168±0.013 | -20.5±1.32 | */* | */* |
| CUR/GOQDs | 16.12±3.34 | 0.252±0.015 | -23.6±3.24 | 91.38±2.85 | 15.12±0.35 |
| EE: Encapsulation efficiency; PDI: Polydispersity index; | | | | | |
